# Hospice utilization during the SARS outbreak in Taiwan

**DOI:** 10.1186/1472-6963-6-94

**Published:** 2006-12-01

**Authors:** Tzeng-Ji Chen, Ming-Hwai Lin, Li-Fang Chou, Shinn-Jang Hwang

**Affiliations:** 1 Department of Family Medicine, Taipei Veterans General Hospital, Shih-Pai Road, Section 2, No 201, Taipei, 11217 Taiwan; 2 National Yang-Ming University School of Medicine, Taipei, Taiwan; 3 Department of Public Finance, National Chengchi University, Taipei, Taiwan

**Keywords:** Severe Acute Respiratory Syndrome, Severe Acute Respiratory Syndrome, Hospice Care, National Health Insurance Research Database, Severe Acute Respiratory Syndrome Patient

## Abstract

**Background:**

The severe acute respiratory syndrome (SARS) epidemic threw the world into turmoil during the first half of 2003. Many subsequent papers have addressed its impact on health service utilization, but few have considered palliative (hospice) care. The aim of the present study was to describe changes in hospice inpatient utilization during and after the SARS epidemic in 2003 in Taiwan.

**Methods:**

The data sources were the complete datasets of inpatient admissions during 2002 and 2003 from the National Health Insurance Research Database. Before-and-after comparisons of daily and monthly utilizations were made. Hospice analyses were limited to those wards that offered inpatient services throughout these two years. The comparisons were extended to total hospital bed utilization and to patients who were still admitted to hospice wards during the peak period of the SARS epidemic.

**Results:**

Only 15 hospice wards operated throughout the whole of 2002 and 2003. In 2003, hospice utilization began to decrease in the middle of April, reached a minimum on 25 May, and gradually recovered to the level of the previous November. Hospices showed a more marked reduction in utilization than all hospital beds (*e.g*. -52.5% *vs*. -19.9% in May 2003) and a slower recovery with a three-month lag. In total, 566 patients were admitted to hospice wards in May/June 2003, in contrast to 818 in May/June 2002. Gender, age and diagnosis distributions did not differ.

**Conclusion:**

Hospice inpatient utilization in Taiwan was indeed more sensitive to the emerging epidemic than general inpatient utilization. A well-balanced network with seamless continuity of care should be ensured.

**Electronic supplementary material:**

The online version of this article (doi:10.1186/1472-6963-6-94) contains supplementary material, which is available to authorized users.

## Background

The severe acute respiratory syndrome (SARS) epidemic threw the world into turmoil during the first half of 2003 [1, 2]. Since then, thousands of papers had been devoted to this novel virus, many have addressed its impact on health service utilization [3–8], but few have focused on hospice palliative care [9]. When the fears declined and utilization data became available, we were able retrospectively to review the broad hospice situation during the SARS epidemic in Taiwan, one of the most heavily affected countries.

The first inpatient hospice ward in Taiwan was established in 1990 [10]. By the end of 2004, there were 26 hospice wards with 424 beds and 42 units for hospice home care [11]. All hospice care programs in Taiwan until then had been hospital-based. A universal health insurance program started in Taiwan in 1995 and now covers nearly all inhabitants (21,984,415 beneficiaries at the end of 2003, equivalent to a coverage rate of 97.3%) [12]. During the early years of National Health Insurance (NHI), admission to hospice wards was reimbursed on a fee-for-service basis, as in acute wards. The payment for services of nurses and other ancillary staff was included in the room fee. This method of reimbursement put the intensive-care hospice at a great disadvantage. After years of negotiation, the Bureau of National Health Insurance began to implement a special program for hospice wards in July 2000. The payment changed to a per-diem lump sum and was generally improved. Any hospice ward was free to join the new program, which also had stricter requirements on personnel and facilities.

The first case of SARS in Taiwan was identified on 14 March 2003 [13]. Until 21 April, only 28 probable SARS cases were reported. After 22 April, the incidence abruptly increased and was associated primarily with health-care settings. Moreover, in many cities and regions of Taiwan, the infection spread from one hospital to several others. Five hospitals had to discontinue routine or emergency services totally. Stricter quarantine was implemented after 28 April. Real panic ensued in Taiwanese society. The last probable case was reported on 14 June, and Taiwan was removed from the list of SARS-affected countries by the World Health Organization on 5 July 2003 [14]. In retrospect, the SARS epidemic in Taiwan had involved 668 probable cases with severe deterioration of pulmonary function and 181 deaths [15].

The aim of the current study was to describe the changes in hospice inpatient utilization during and after the SARS epidemic within the NHI program in Taiwan. Such quantitative analyses might provide evidence for discussion in health policy-making and help with effective planning in the future.

## Methods

### Data sources

In 1999, the Bureau of National Health Insurance began to release all claims data to the public in electronic form under the National Health Insurance Research Database (NHIRD) project [16]. Since then, dozens of extracted datasets have been available to researchers. The identification numbers of persons and healthcare facilities in the datasets have been encrypted to protect privacy. The encryption is consistent across all datasets so that longitudinal comparisons are feasible. However, no linkage with external databases and medical records was possible. Researchers who wish to access NHIRD datasets must sign a user agreement form indicating that they will obey relevant regulations and acknowledge the NHIRD in their publications.

In the current study, we used the complete inpatient admission datasets from 2000 to 2003 (DD{2000..2003} {01..12}.DAT). Each record in the admission file contains the data for one admission, including the re-coded identification numbers of the patient and hospital, type of benefits, dates of admission and discharge, five discharge diagnoses, and items of expenditure. We also used the registry for contracted medical facilities (HOSB2003.DAT) to identify the accreditation levels and locations of the hospitals with hospice wards, and the registry for contracted beds (BED2003.DAT) to compute the number of beds in each hospice ward.

### Study design

To begin with, we extracted the admission records for hospice wards according to the type of benefit. Twenty-five hospitals had participated in the NHI hospice program: from 12 hospitals in July 2000 to 21 in December 2003, during which time 4 hospitals dropped out. After computing the utilization of hospice beds for each day, we found that there were indeed cyclic fluctuations around the year. Because data were only available for a few years and the numbers of hospice wards and beds had increased over time, it did not seem appropriate to deploy a conventional time series analysis, *e.g*. the autoregressive integrated moving average method [3, 4, 17]. Instead, we performed a before-and-after comparison of monthly utilizations between 2002 and 2003 and plotted daily utilization. The analysis was limited to those hospitals that offered hospice inpatient services throughout the whole of 2002 and 2003. Because insurance claims were normally submitted after the patients' discharge, the claims for the final month of the available datasets contained no data for patients who were still hospitalised at the end of that month. Thus, the data for December 2003 were not analysed.

We extended the before-and-after comparison to the totality of hospital beds within the National Health Insurance to contrast with the utilization of hospice wards. To indicate the situation in individual hospice wards, we computed the changes of utilization in each hospice ward separately during and after the SARS epidemic. These data were further stratified by the accreditation levels and locations of the hospice wards. We also analysed the patients who were admitted to hospice wards during the peak period of the SARS epidemic (*i.e*. May/June 2003) and compared them with the patients admitted during the same months of the previous year. This latter analysis included age, sex, and primary diagnosis at discharge and was also stratified by ward accreditation level and location.

### Data processing and statistical analysis

The Perl programming language (version 5.8.7) was used for the extraction and computation of data [18]. The unit of hospice/hospital ward utilization was the patient-day, *i.e*. each claimed day of hospitalisation. Conventionally, the discharge day is not counted as a hospitalisation day in insurance claims. For example, if a patient was hospitalised from 1 January to 7 January, the per-diem fees for physician and room services were payable only from 1 January to 6 January. We used two-sample *t*-tests to compare the age distributions of those admitted in May/June 2003 with those admitted in May/June 2002, and Pearson's chi-square test to compare the sex and diagnosis distributions. We also used one-way analysis of variance to compare the age distributions of those admitted in May/June 2003 to hospice wards of different accreditation levels and locations. A *P*-value < 0.05 was regarded as statistically significant. The statistical analyses were performed using SPSS software (Release 13.0 for Windows, 2004, SPSS Inc., Chicago).

## Results

**Figure 1:**
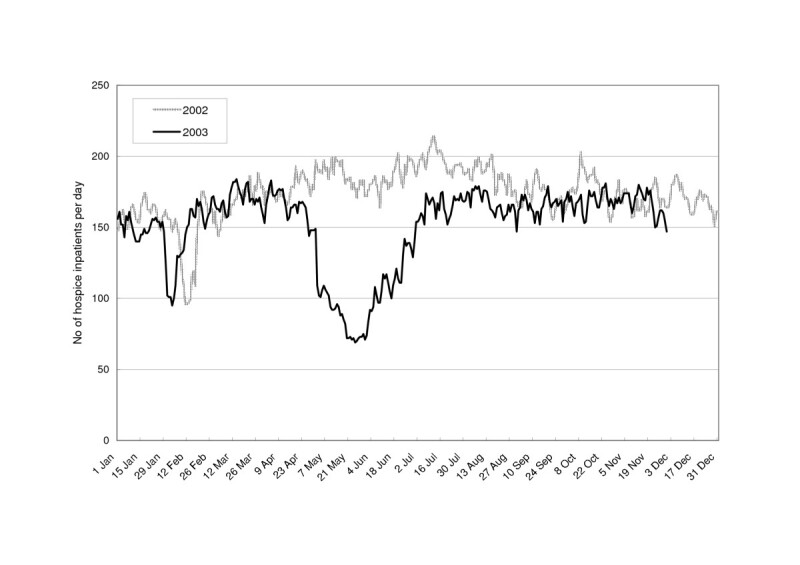
Number of patients per day in 15 hospice wards during 2002 (non-SARS year) and 2003 (SARS year).

**Figure 2:**
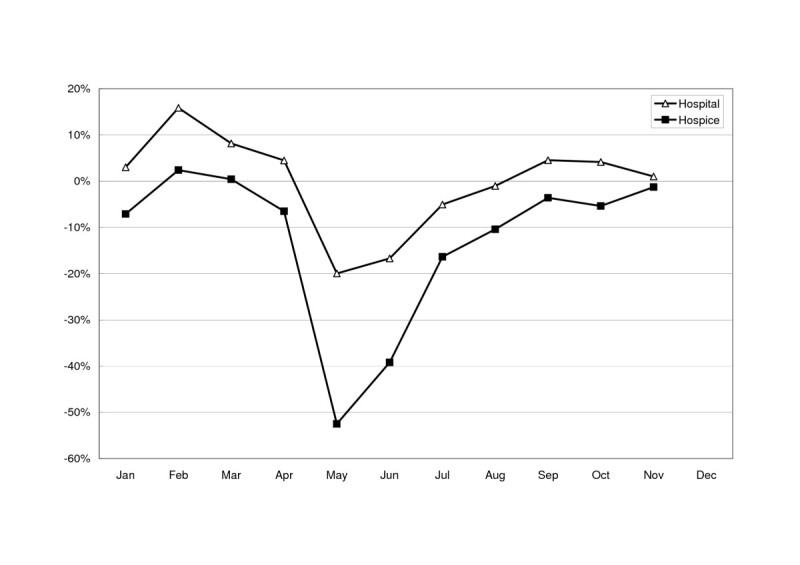
Differences between aggregate patient-days of hospice beds and all kinds of hospital beds in 15 hospitals during each month of 2003 compared with the corresponding months of 2002. Difference = ((patient-days in a month of 2003) – (patient-days in a month of 2002))/(patient-days in a month of 2002).

Only 15 hospitals in Taiwan offered hospice inpatient services throughout the whole of 2002 and 2003. The numbers of patients in these wards on each day of 2002 and 2003 are shown in Figure 1. In 2002, the maximal capacity was 214 patients per day (on 11 July) and the regular utilization was between 150 and 200 patients per day. Two noticeable troughs in February 2002 and January 2003 corresponded with the Chinese New Year holidays, which are based on the lunar calendar. In 2003, hospice utilization began to decrease in the middle of April, reached a minimum on 25 May (69 patients), and gradually recovered to the level of the previous November. The trend in utilization over 15 hospice wards during 2003 was parallel to that in all hospitals, except that the hospice wards showed a more marked reduction in utilization during the peak period of the SARS epidemic (-52.5% *vs*. -19.9% in May 2003) and a slower recovery with a three-month lag (Figure 2).

The influence of the SARS epidemic on hospice utilization varied widely among the 15 wards studied (Table 1). Even during the peak period of the epidemic, three wards showed growth in utilization and one was unchanged, while two seemed temporarily to cease operating. The recovery also varied from ward to ward: two months after the epidemic, four wards still showed a reduction in utilization of more than 20%.

**Table 1:** Differences between bimonthly patient-days in individual hospice wards during 2003 and those at the same periods in 2002

Hospice	March/April	May/June	July/August	September/October
A	-5%	-98%	-59%	-31%
B	-8%	-94%	-39%	-7%
C	19%	-64%	-20%	-21%
D	-22%	-57%	-21%	-27%
E	-8%	-50%	-9%	3%
F	20%	-45%	2%	8%
G	-3%	-44%	-25%	-9%
H	0%	-44%	0%	-2%
I	-11%	-41%	-39%	-37%
J	7%	-15%	20%	-13%
K	2%	-5%	5%	27%
L	-18%	0%	-4%	1%
M	-11%	5%	-1%	9%
N	-10%	6%	7%	-4%
O	32%	15%	51%	32%
Total	-3%	-46%	-13%	-4%

*Difference = ((patient-days in two months of 2003) – (patient-days in two months of 2002))/(patient-days in two months of 2002).

In total, 566 patients were admitted to hospice wards in May/June 2003, in contrast to 818 admissions in May/June 2002. The gender, age and diagnosis distributions did not differ between patient groups in these two years (Table 2).

**Table 2:** Characteristics of hospice patients admitted during the peak period of the severe acute respiratory syndrome and one year earlier

	Admission in May/June 2002 (*N* = 818), *n* (%)	Admission in May/June 2003 (*N* = 566), *n* (%)	*P* value
Gender			0.669*
Female	352 (43.0)	249 (44.0)	
Male	465 (56.8)	317 (56.0)	
Unknown	1 (0.1)		
Age in years	64.6 (SD 14.6)	64.6 (SD 15.0)	0.946**
Primary diagnosis			0.981*
Ca of trachea, bronchus, & lung	147 (18.0)	98 (17.3)	
Ca of liver & intrahepatic bile ducts	140 (17.1)	93 (16.4)	
Ca of colon	56 (6.8)	37 (6.5)	
Ca of female breast	48 (5.9)	40 (7.1)	
Ca of stomach	51 (6.2)	30 (5.3)	
Ca of pancreas	37 (4.5)	24 (4.2)	
Ca of rectum, rectosigmoid junction, & anus	32 (3.9)	21 (3.7)	
Ca of cervix uteri	29 (3.5)	19 (3.4)	
Others	278 (34.0)	204 (36.0)	

*Pearson's chi-square test, two-sided.

**Two-sample *t*-test, two-tailed.

Of the 15 wards, eight were located in academic medical centres, six in metropolitan hospitals, and one in a local community hospital. As to geographic location, six were located in northern Taiwan, two in the middle of the country, six in the south and one in the east (Table 3).

**Table 3:** Distribution of hospice wards by accreditation level and geographic location (aggregate numbers of hospice beds in parentheses)

	Academic medical centre	Metropolitan hospital	Local community hospital	Total
Northern Taiwan	4 (110)	2 (27)		6 (137)
Middle Taiwan	1 (12)	1 (10)		2 (22)
Southern Taiwan	2 (29)	3 (38)	1 (13)	6 (80)
Eastern Taiwan	1 (15)			1 (15)
Total	8 (166)	6 (75)	1 (13)	15 (254)

**Figure 3:**
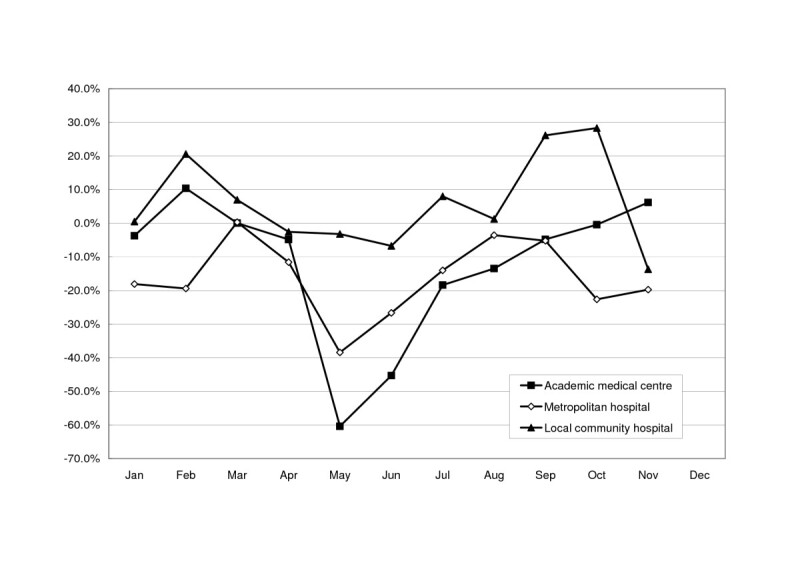
Differences between aggregate patient-days in hospice wards during each month of 2003 and those of the corresponding months of 2002, stratified by accreditation level. Difference = ((patient-days in a month of 2003) – (patient-days in a month of 2002))/(patient-days in a month of 2002).

**Figure 4:**
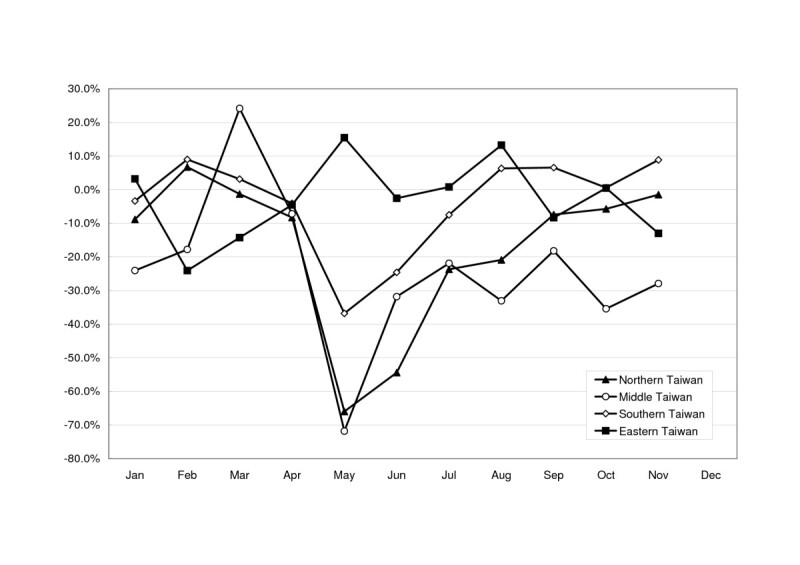
Differences between aggregate patient-days in hospice wards during each month of 2003 and those of the corresponding months of 2002, stratified by geographic location. Difference = ((patient-days in a month of 2003) – (patient-days in a month of 2002))/(patient-days in a month of 2002).

During the peak period of the SARS epidemic, hospice wards in academic medical centres were affected more severely than those in metropolitan hospitals (-60.4% *vs*. -38.4% in May 2003), whereas the one in a local community hospital was only slightly affected (-3.2% in May 2003) (Figure 3). Hospice wards in northern and middle Taiwan were affected more severely than those in southern Taiwan (-66.0% and -71.8% *vs*. -36.8% in May 2003), whereas the one in eastern Taiwan showed increased utilization (+15.4% in May 2003) (Figure 4).

Among the 566 patients admitted to hospice wards during the peak of the epidemic, the gender and age distributions did not differ among patient groups in academic medical centres, metropolitan hospitals or the local community hospital (Table 4). The gender distribution did not differ among patient groups in different locations, but the age distribution did (Table 5).

**Table 4:** Characteristics of hospice patients admitted during the peak period (May/June 2003) of the severe acute respiratory syndrome, stratified by accreditation level

	Academic medical centres (*N* = 372), *n* (%)	Metropolitan hospitals (*N* = 165), *n* (%)	Local community hospitals (*N* = 29), *n* (%)	*P* value
Gender				0.582*
Female	159 (42.7)	75 (45.5)	15 (51.7)	
Male	213 (57.3)	95 (54.5)	14 (48.3)	
Age in years	65.2 (SD 15.2)	64.3 (SD 14.3)	59.0 (SD 15.2)	0.091**
Primary diagnosis				
Ca of trachea, bronchus, & lung	63 (16.9)	30 (18.2)	5 (17.2)	
Ca of liver & intrahepatic bile ducts	58 (15.6)	31 (18.8)	4 (13.8)	
Ca of colon	26 (7.0)	10 (6.1)	1 (3.4)	
Ca of female breast	23 (6.2)	13 (7.9)	4 (13.8)	
Ca of stomach	21 (5.6)	8 (4.8)	1 (3.4)	
Ca of pancreas	17 (4.6)	6 (3.6)	1 (3.4)	
Ca of rectum, rectosigmoid junction, & anus	16 (4.3)	4 (2.4)	1 (3.4)	
Ca of cervix uteri	10 (2.7)	7 (4.2)	2 (6.9)	
Others	138 (37.1)	56 (33.9)	10 (34.5)	

*Pearson's chi-square test, two-sided.

**One-way analysis of variance, *F (df)* = 2.403 (2, 563).

**Table 5:** Characteristics of hospice patients admitted during the peak period (May/June 2003) of the severe acute respiratory syndrome, stratified by location

	Northern Taiwan (*N* = 225), *n* (%)	Middle Taiwan (*N* = 35), *n* (%)	Southern Taiwan (*N* = 248), *n* (%)	Eastern Taiwan (*N* = 58), *n* (%)	*P* value
Gender					0.311*
Female	106 (47.1)	11 (31.4)	109 (44.0)	23 (39.7)	
Male	119 (52.9)	24 (68.6)	139 (56.8)	25 (60.3)	
Age in years	66.6 (SD 14.7)	61.6 (SD 16.7)	62.5 (SD 14.8)	67.9 (SD 14.3)	0.005**
Primary diagnosis					
Ca of trachea, bronchus, & lung	42 (18.7)	5 (14.3)	43 (17.3)	8 (13.8)	
Ca of liver & intrahepatic bile ducts	38 (16.9)	4 (11.4)	42 (16.9)	9 (15.5)	
Ca of colon	12 (5.3)	1 (2.9)	18 (7.3)	6 (10.3)	
Ca of female breast	17 (7.6)		20 (8.1)	3 (5.2)	
Ca of stomach	13 (5.8)	1 (2.9)	15 (6.0)	1 (1.7)	
Ca of pancreas	7 (3.1)	4 (11.4)	10 (4.0)	3 (5.2)	
Ca of rectum, rectosigmoid junction, & anus	9 (4.0)	1 (2.9)	8 (3.2)	3 (5.2)	
Ca of cervix uteri	3 (1.3)		14 (5.6)	2 (3.4)	
Others	84 (37.3)	19 (54.3)	78 (31.5)	23 (39.7)	

*Pearson's chi-square test, two-sided.

**One-way analysis of variance, *F (df)* = 4.39 (3, 562).

## Discussion

Most studies of the impact of SARS on health service utilization have been confined to a single facility. Our study, based on nationwide data, gave a complete and exact picture of hospice utilization. The hospice, also known as palliative care, has usually been considered as conservative and non-acute. One could well imagine that the use of hospice services would be compromised because of fears during uncertain times. Our findings support this view. Hospice inpatient utilization in Taiwan was indeed more sensitive to the emerging epidemic than general inpatient utilization. Not only was the reduction sharper, but also recovery to the previous level took longer.

Although it cannot be classed as acute care, hospice care is unlike elective surgery, which can put patients on a long waiting list. Hospice care is provided to terminally ill patients whose life expectancy is limited. During the SARS epidemic in Taiwan, many terminally ill patients might have been suspended or deterred from professional hospice care because well-organized patient transfers did not exist. Health consequences might not be a big issue among hospice patients. Instead, quality of care and humanity are the major concerns.

During the peak period of the SARS epidemic, *i.e*. May/June 2003, the number of admissions to the 15 hospice wards in our study decreased to 69% of those in the previous year, and the utilization in patient-day units to 54%. This meant that some patients might have been discharged earlier. It remains unknown whether the decrease of utilization was due to patients' voluntary decisions or to manoeuvring by the hospitals. According to our personal experiences, both patients' decisions and hospital policies could have played a role. On the patient's side, the family might have a major part in the decision about hospice care, because weak terminally ill patients are usually dependent on their families. The fear of SARS might have come from the family rather than the patient. Only interviews with the patients and their caregivers could give a satisfactory answer.

The aggregate utilization of the 15 hospice wards during the peak period of the SARS epidemic in Taiwan was lower than that during the Chinese New Year holidays (Figure 1). Like Christmas to Christians, the Chinese New Year holidays are important to most Chinese as occasions of family reunion. Usually hospital activities are kept to a minimum on these days. The daily curve of hospice utilization vividly illustrated the impact of the SARS epidemic.

As for the patients admitted to hospice wards during the SARS epidemic, the insurance claims used in our study could only offer three reliable variables (gender, sex and discharge diagnoses), which showed no differences. Because about 60% of cancer patients in Taiwan die at home [19], we could not calculate the survival time of every hospice patient as a proxy of disease severity from our closed datasets.

Although Taiwan had been declared a SARS-affected country, not every hospice ward was equally influenced by this epidemic. The impact was related to the locations and accreditation levels of the hospitals with hospice wards: the bigger the hospital, the stronger the SARS impact. Other features, *e.g*. the admission policy and mission of each hospital during the SARS epidemic, might have played a role. Hospices in middle Taiwan were most severely affected; only the hospice in eastern Taiwan was negligibly affected. The main island of Taiwan has 22 cities and counties. The probable cases of SARS appeared mainly in Taipei city (in northern Taiwan), Taipei county (northern), Taoyuan county (northern), Kaohsiung city (southern) and Kaohsiung county (southern) [13]. On 26 March 2003, a resident of Hong Kong's Amoy Gardens flew to Taiwan and travelled to Taichung (in middle Taiwan). That man's brother in Taiwan became Taiwan's first SARS fatality [15]. This might partly explain the impact of SARS in middle Taiwan (Figure 4).

Because the NHIRD prohibited any break of confidentiality, we did not continue the analysis hierarchically from geographic location to accreditation level. Instead, we displayed the aggregate data in location and level separately. However, hierarchical modelling could be used to examine the factors associated with reduced utilization and would help construct a real-time and on-line analytical processing system for nationwide surveillance during an outbreak of an infectious disease.

A striking feature of the SARS epidemic in Taiwan was that 56.3% of SARS patients were hospital-related [15]. In retrospect, it was reasonable and preferable to reduce hospitalisations. A similar phenomenon was observed simultaneously in childbirth in Taiwan [7]. Many deliveries were shifted from secondary and tertiary care hospitals to primary clinics. The question remains whether continuity and quality of care could be ensured. It is unlikely that patients shifted from the 15 hospices in the current study to the other six (unanalysed) hospices, which either quitted or joined the hospice program after the SARS epidemic. However, the possibility of patient flow to other non-hospice wards cannot be excluded. Our claims-based study could not determine whether the needs of patients with terminal illnesses were met during the epidemic, and threw no light on the psychological impact on patients and healthcare workers [20].

## Conclusion

The SARS epidemic will not be the last to affect humankind. The lessons for the hospice society in Taiwan and for the world might be to distribute services into well-balanced networks, *e.g*. hospice care at home (through visits by physicians and nurses), shared hospice care in hospitals (*i.e*. providing hospice care in non-hospice wards), and hospice care at independent hospices in the community. A seamless continuity of care between facilities should be ensured at all times.

## Authors’ original submitted files for images

Below are the links to the authors’ original submitted files for images. Authors’ original file for figure 1
Authors’ original file for figure 2
Authors’ original file for figure 3
Authors’ original file for figure 4

## Abbreviations

NHI: National Health Insurance
NHIRD: National Health Insurance Research Database
SARS: severe acute respiratory syndrome
