# Supplementary figures and images for: Hospice utilization during the SARS outbreak in Taiwan
Source: BMC Health Serv Res. 2006 Dec 1;6:94. doi: 10.1186/1472-6963-6-94 (PMC1559606; doi:10.1186/1472-6963-6-94)

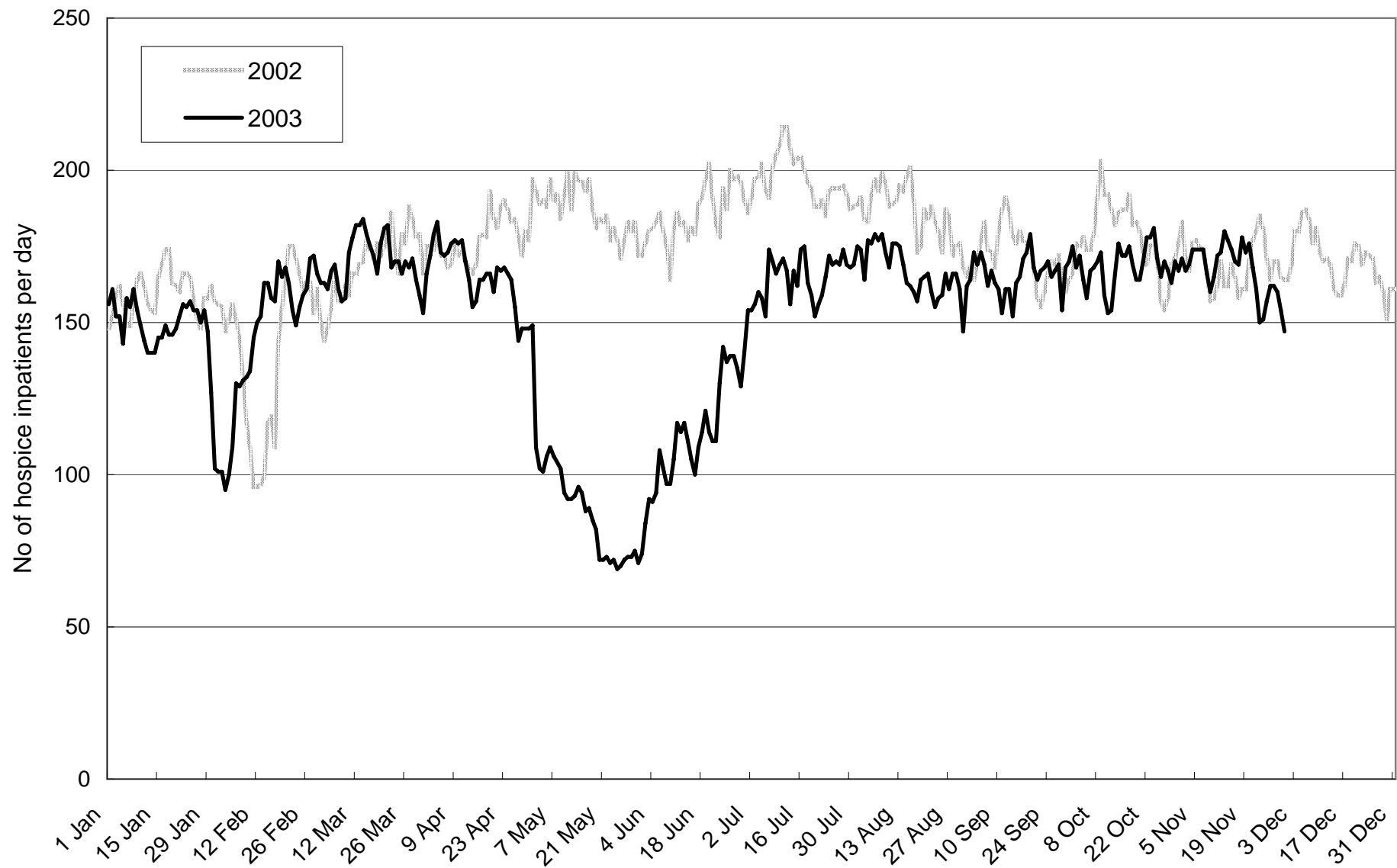

Supplement: Supplementary file 1 — Authors’ original file for figure 1 [file 12913_2006_271_MOESM1_ESM.pdf]

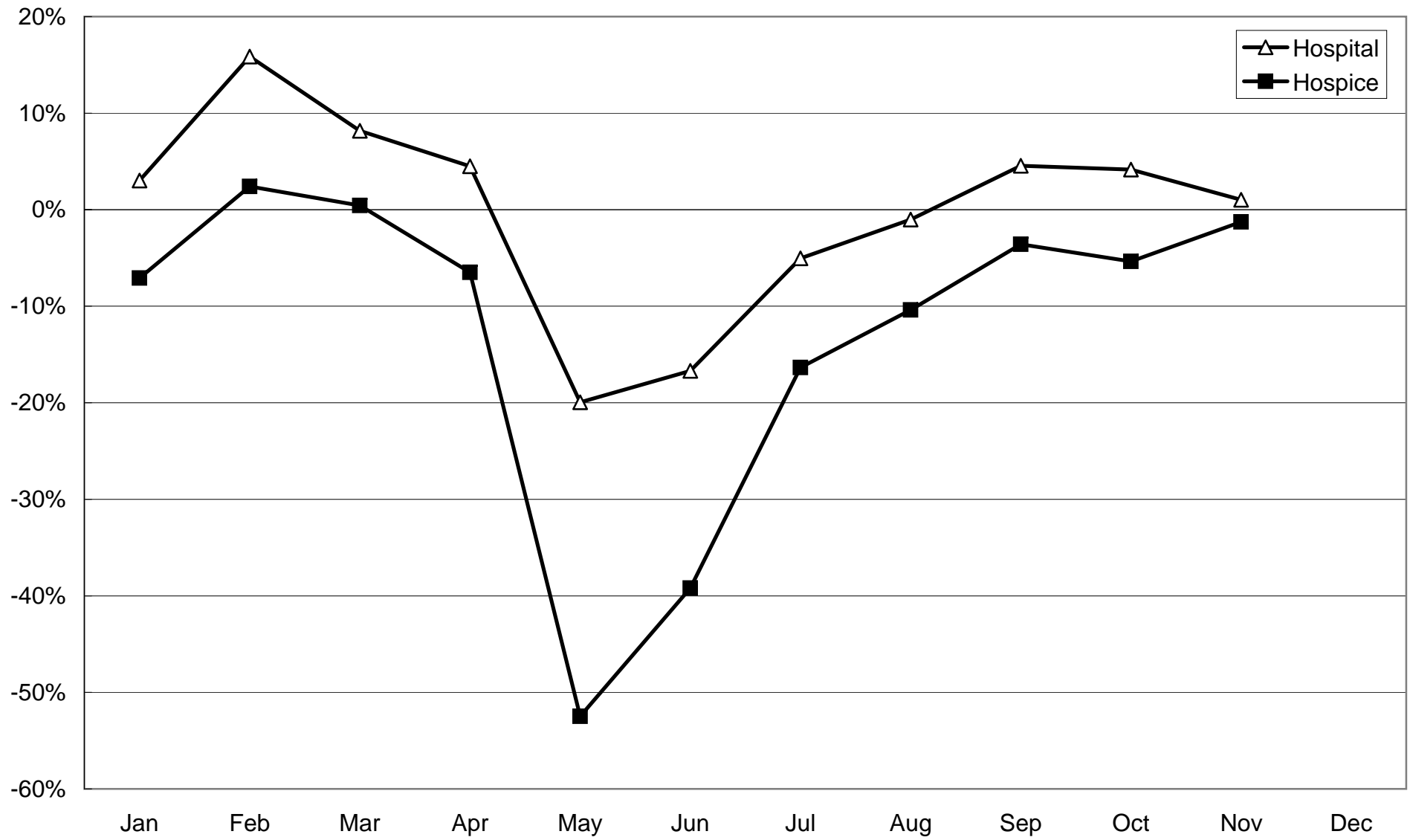

Supplement: Supplementary file 2 — Authors’ original file for figure 2 [file 12913_2006_271_MOESM2_ESM.pdf]

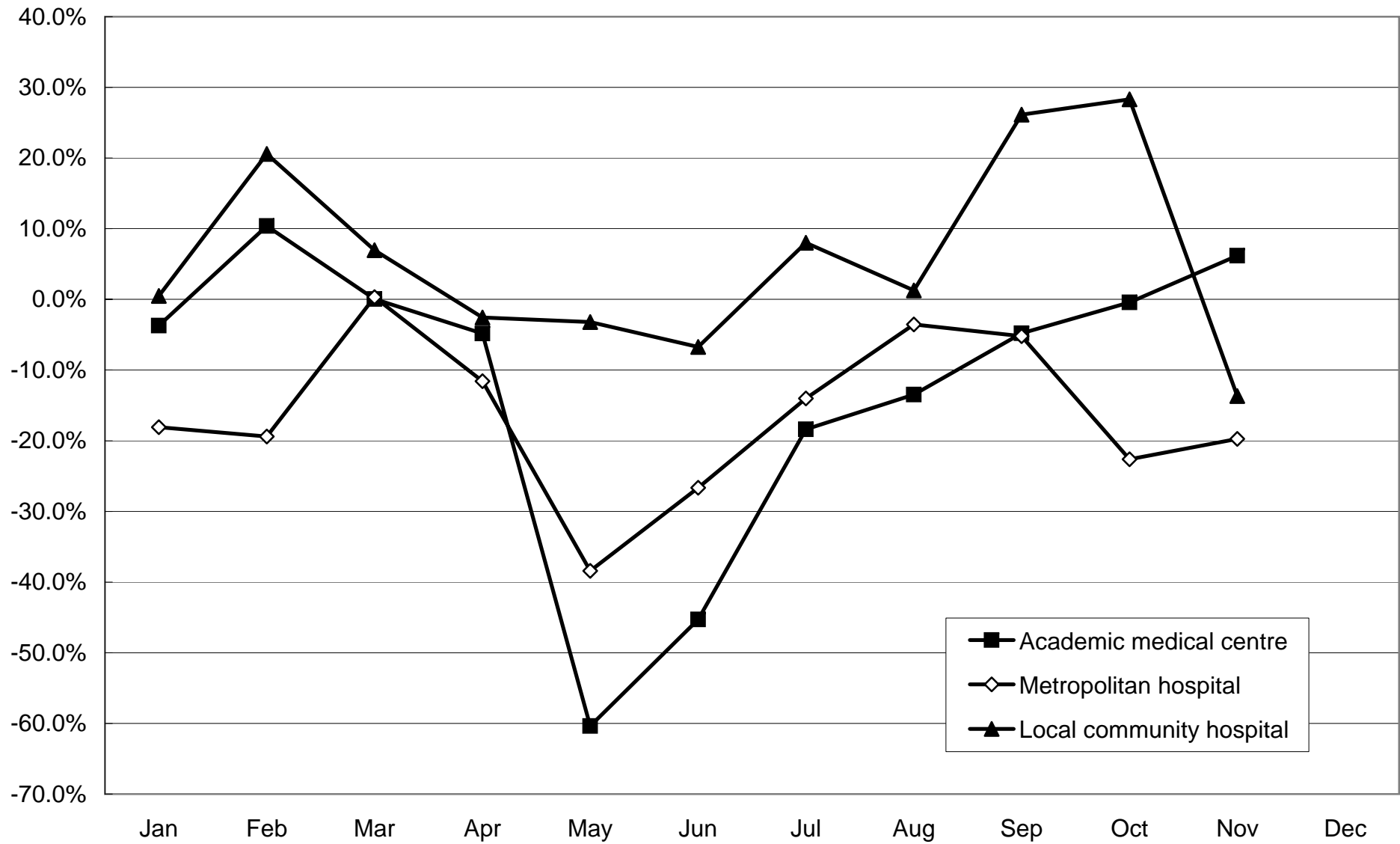

Supplement: Supplementary file 3 — Authors’ original file for figure 3 [file 12913_2006_271_MOESM3_ESM.pdf]

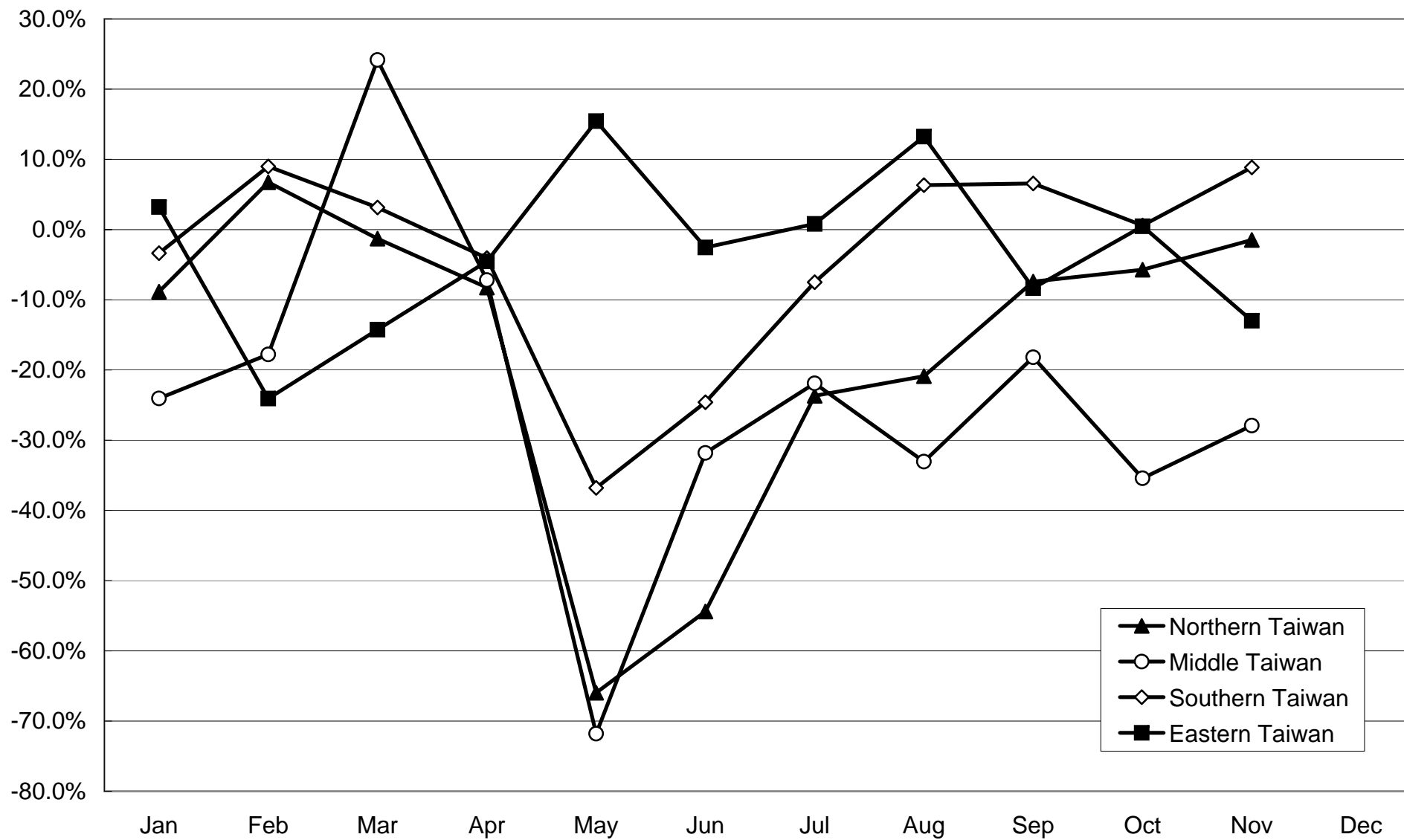

Supplement: Supplementary file 4 — Authors’ original file for figure 4 [file 12913_2006_271_MOESM4_ESM.pdf]
